# Rupture prématurée des membranes à terme: facteurs pronostiques et conséquences néonatales

**DOI:** 10.11604/pamj.2017.26.68.11568

**Published:** 2017-02-05

**Authors:** Asmama Yasmina, Amina Barakat

**Affiliations:** 1 Unité de Soins et Réanimation Néonatale, Service de Pédiatrie V, Hôpital d’Enfants de Rabat, CHU Ibn Sina, Maroc

**Keywords:** Rupture prématuré, à terme, infection materno-fœtale, Prelabour rupture, at term, maternal-fetal infection

## Abstract

La rupture prématurée des membranes (RPM) à terme survient dans 5 à 10% des grossesses. Elle rend compte d'une part importante de morbidité et de mortalité néonatales. Le but de cette étude est de déterminer les facteurs pronostiques maternels et obstétricaux ainsi que le devenir des nouveau-nés à terme issus d'une grossesse compliquée de rupture prématurée des membranes ayant été hospitalisés au service ou gérés à la consultation externe. Etude rétrospective, analysant toutes les observations de nouveau-nés à terme issus de grossesses compliquées de RPM, enregistrées au service de néonatologie de l'hôpital d'enfants de Rabat entre le 1^er^ janvier et le 31 juillet 2014. Au cours de la période d'étude nous avons colligé 144 cas de RPM isolée sur un total de 2400 naissances vivantes (NV) soit une prévalence de 6% NV, répartis comme suit : 06 cas de RPM (4%) entre 6 et 12 heures, 14 cas (9,7%) entre 12 et 18 heures, 28 cas (19,4%) entre 18 et 24 heures et 96 cas (66,6%) supérieure à 24 heures. La majorité de nos parturientes étaient dans la tranche d'âge de 25 à 35ans avec un taux de 52%. Une chorioamniotite associée a été retenue dans 8,3% des cas. Les parturientes ont été mises sous antibiothérapie prophylactique par voie orale ou parentérale dans 28% des cas, avec un liquide amniotique clair dans 81% des cas. Le diagnostic d'IMF probable a été retenu dans 46 cas dont 65.2 % dans le sous groupesupérieur à 18 h versus respectivement 26% et 8,7% dans les sous groupe 12 à 18 h et inférieur à 12 h. A l'admission, on a noté une prédominance masculine avec 58,3%, les nouveau-nés étaient asymptomatiques dans 76% des cas et ils présentaient une détresse respiratoire dans 42,8% des cas, un ictère dans 31,45% des cas, une fièvre dans 14,2% des cas et des signes de souffrance neurologique dans 11,5% des cas. Tous les nouveau-nés hospitalisés, soit dans 72% des cas, ont été mis sous antibiothérapie pendant une durée allant de 5 à 10 jours avec une durée d'hospitalisation moyenne de 2,44jours. Ce travail souligne le risque important d'IMF associé à une RPM même à terme. Ce risque est d'autant plus important que la rupture est supérieure à 24 heures de temps. Dans la majorité des cas le liquide amniotique est clair et les nouveau-nés sont asymptomatiques à l'admission, ce qui laisse la mise sous antibiothérapie automatiquement de ces nouveau-nés un sujet encore très discutable.

## Abstract (en)

Prelabour rupture of membranes (PROM) at term occurs in 5 to 10% of pregnancies. It accounts for a significant proportion of neonatal morbidity and mortality. The aim of this case study was to determine the maternal and obstetric prognostic factors as well as full-term newborns outcomes in pregnancies complicated by prelabour rupture of membranes in patients hospitalized or managed at the outpatient clinic. We conducted a retrospective study of all cases of full-term infants born to mothers whose pregnancy was complicated by PROM, recorded in the neonatology department at the Children’s Hospital of Rabat between 1 January and 31 July 2014. During the study period we collected 144 cases of PROM isolated from a total of 2,400 live births (LB), ie a prevalence of live births (6%), distributed as follows: 6 cases of PROM (4%) between 6 and 12 hours, 14 cases (9.7%) between 12 and 18 hours, 28 cases (19.4%) between 18 and 24 hours and 96 cases (66.6%) of more than 24 hours. The majority of parturients were within the age-group 25-35 years with a rate of 52%. The diagnosis of associated chorioamniotitis was retained in 8.3% of cases. Parturients were treated with oral or parenteral antibiotic prophylaxis in 28% of cases with clear amniotic fluid in 81% of cases. The diagnosis of probable MFI was retained in 46 cases, 65.2% in the subgroup > 18 h versus 26% and 8.7% in subgroups 12-18 h and <12 h respectively. On admission, there was a male predominance of 58.3%, newborns were asymptomatic in 76% of cases, they suffered from respiratory distress in 42.8% of cases, jaundice in 31.45% of cases, fever in 14.2% of cases and signs of neurological distress in 11.5% of cases. All hospitalized newborns (72% of cases), were treated with antibiotics for a period ranging from 5 to 10 days with an average hospital stay of 2.44 days. This case study highlights the significant risk of MFI associated with PROM even in pregnant woman at term. This risk is major when rupture of membranes occurs after 24 hours of time. In the majority of cases the amniotic fluid is clear and newborns are asymptomatic on admission, leaving antibiotic therapy in these newborns a controversial subject.

## Introduction

La rupture prématurée des membranes (RPM) est définit par une ouverture avérée de la poche des eaux avant tout début de travail. Elle complique 2 à 3% des grossesses, 1/3 des naissances prématurées et 60 à 80% des grossesses après 37 SA. Cette pathologie est responsable de complications maternelles et fœtales, et ces complications néonatales sont de types respiratoires, neurologiques ou infectieuses. Nous avons menée une étude rétrospective dans le service de réanimation néonatale de l'hôpital d'enfants de Rabat sur une durée de 07mois allant de Janvier à Juillet 2014, portant sur les nouveau-nés nés à terme avec une notion de RPM et ayant été hospitalisés au service ou gérés à la consultation externe. Le but de notre travail était de définir les facteurs pronostiques anténatals et postnatals susceptibles d'influencer le devenir de ces nouveau-nés et d'évaluer leur évolution à court et moyen terme.

## Case study

Sur une durée de 07mois allant de Janvier à Juillet 2014, nous avons colligés 144 naissances à terme à la maternité ou référées vers notre service pour prise en charge d'une rupture prématurée des membranes avérée avant le travail. Les nouveau-nés ont été gérés en hôpital du jour à la consultation ou hospitalisés dans notre service pour une durée allant de 2 à 10 jours. Les données épidémiologiques ont été recueillies à partir des dossiers médicaux et des registres des naissances de la maternité. Les critères d'inclusion étaient : tout nouveau-né à terme symptomatique avec notion de RPM quelque soit sa durée et toute RPM >18 heures avec CRP à H24 faite systématiquement. Les critères d'exclusion étaient : tout nouveau-né né avant 37SA et les RPM de durée inférieure à 4heures. L'évaluation clinique des nouveau-nés à la naissance portait sur le poids de naissance, le score d'Apgar à une, cinq et dix minutes, sur le statut respiratoire, infectieux et neurologique. Tous les nouveau-nés infectés ont bénéficié d'une triple antibiothérapie par ampicilline, céphalosporine et aminoside conformément aux recommandations, et selon les données des explorations biologiques, bactériologiques et radiologiques.

**Figure 1:**
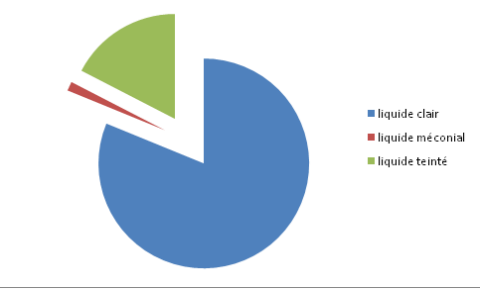
Répartition selon couleur du LA

**Figure 2:**
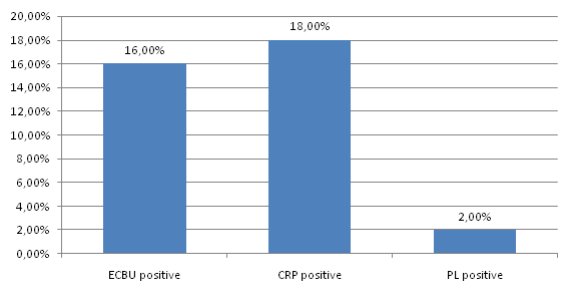
Résultats du bilan infectieux chez le nouveau-né

Dans notre étude, cent quarante quatre cas de rupture prématurée des membranes sur un total de 2400 naissances, ont été constatés soit un taux de 6%. 70 des parturientes étaient primipares, soit dans 48,6% des cas, et 74 autres étaient multigestes. Il y avait une grande variation dans l'âge des femmes enceintes, allant de 19 ans à 48 ans avec un âge moyen estimé à 28,21+/- 6,4 ans. Pour notre part, 63% des patientes appartenaient à la classe sociale démunie, 31% des patientes à la classe sociale moyenne et seulement 6% des patientes appartenaient à la haute classe sociale. Concernant la durée de la rupture des membranes par rapport au début du travail, dans 58,4%des cas la rupture des membranes était prolongée (Tableau 1). La couleur du liquide amniotique était claire dans 81,2% des cas, avec un taux de virage du liquide amniotique estimé à 18,8% (Figure 1). Une chorioamniotite associée a été documentée chez 12 des parturientes, soit dans 8,3% des cas. Les parturientes ont été mise sous antibiothérapie prophylactique avant l'accouchement dans 28% des cas, et ceci devant un bilan infectieux positif dans seulement 18% des cas. Il s'agissait d'une triple antibiothérapie faite d'une ampicilline avec un aminoside et un anti-anaérobie dans 38% des cas, d'une monothérapie par amoxicilline simple dans 62% des cas. L'accouchement était par voie basse dans 72,2% des cas, par voie haute dans 27,8% des cas. Le motif de la césarienne était dans 50% des cas un échec de déclenchement, une présentation anormale dans 20% des cas, pour virage de liquide amniotique dans 17,5% des cas ; ou alors il s'agissait d'une césarienne programmée dans 12,5% des cas et ceci pour bassin limite ou dépassement de terme avec souffrance fœtale aigue (Tableau 2). Dans le groupe des nouveau-nés inclus dans notre étude, nous avons retrouvé une prédominance masculine, avec un pourcentage de 58,3%. Les nouveau-nés à terme avaient un poids <2500g en cas de RCIU en rapport avec une pathologie maternelle à type de pré-éclampsie ou HTA gravidique, ou une pathologie fœtale malformative ou infectieuse. Les nouveau-nés nés à terme étaient asymptomatiques dans 76% des cas, et symptomatiques lors de leur admission au service dans 24% des cas. La symptomatologie était variée et multiple (Tableau 3). Une fois hospitalisés dans notre service, tous les nouveau-nés ont bénéficié d'un bilan biologique fait d'une numération formule sanguine (NFS) et d'une CRP faits après 24heures de vie, avec prélèvements bactériologiques fait de : ECBU, ponction lombaire et hémoculture, ce bilan est revenu positif dans 36% des cas (Figure 2). Dans notre série, l'analyse comparative entre les différents sous groupes de RPM établis a montré que le sous groupe “RPM supérieure à 18 heures “est le plus lié à un risque d'IMF certaine (73%) et d'IMF probable (65,2%). Dans chacun des sous-groupes “6-12 h” et “12-18h” le risque d'IMF probable a été de 8,7% et 26% respectivement (Tableau 4). Tous les nouveau-nés hospitalisés ont été mis sous triple antibiothérapie par voie veineuse, avec une durée d'hospitalisation minimum de 48heures dans notre service, ou gérés uniquement à l'hôpital de jour dans 28% des cas. Une fois hospitalisé, 15% des nouveau-nés ont séjourné en unité de réanimation, en unité de soins intensifs dans 35% des cas et en unité de néonatologie dans 50% des cas. La durée moyenne d'hospitalisation allait de 2 à 10 jours avec une moyenne de 2,44 jours. Aucun cas de décès n'a été noté dans notre étude, avec un taux de séquelles neurologiques et respiratoires nul. L'évolution à moyen terme soit sur une durée de 6mois après leur prise en charge initial était satisfaisante, avec un bon développement staturo-pondéral et psycho-moteur.

**Tableau 1:** Répartition selon la durée de la RPM

Durée RPM	Effectif	Pourcentage
< 24heures	48	34%
24 à 47h	56	38,8%
48 à 72h	30	20,8%
>72heures	10	7%

**Tableau 2:** Caractéristiques épidémiologiques population

Paramètre/RPM	<12h	12-18h	>18h
Age (an)	31,5 (2,6)	27,5 (2,8)	28,4 (3,4)
Accouchement (B/H)	04/03	21/03	78/34
Sexe (M/F)	02/05	13/10	69/45
Apgar (1/5)	7/8	8/9	8/9

**Tableau 3:** Symptomatologie néonatale lors des RPM

Données néonatales	6 à 12h	12 à 18h	>18h	Total
Sexe feminine	05	10	45	60
Sexe masculine	02	13	69	84
Asymptomatique	05	18	86	109
DR	02	05	08	15
Fièvre	00	02	03	05
Détresse neuro	00	01	03	04
Ictère précoce	01	03	07	11

**Tableau 4:** Diagnostic retenu en fonction des sous-groupes de RPM

Données / durée RPM	6 à 12h	12 à 18h	>18h	Total
ECBU positif	02	05	16	23
CRP positive	05	06	15	26
PL positive	00	01	02	03
IMF probable	04	12	30	46
IMF certaine	01	06	19	26

## Discussion

La rupture prématurée des membranes à terme est définit par l'ouverture de la poche des eaux avant le début du travail. Le terme prématuré s'applique au début du travail et non à l'âge gestationnel. On distingue la RPM avant le terme, c´est-à-dire entre 14 et 37 semaine d'aménorrhée(SA), et la RPM à terme survenant à partir de 37SA [1, 2]. La RPM survient pour 5 à 10% des grossesses à terme. Les principaux risques fœtaux sont ceux de l'infection materno-fœtale, de l'oligoamnios et de la prématurité en cas de RPM avant terme. L'une des principales complications de la RPM est la chorio-amniotite ou l'infection materno-fœtale, dont la définition biologique, clinique et histologique reste variable dans la littérature. Ce risque infectieux est variable avec l'âge gestationnel lors de la rupture des membranes. Il est de 5% à terme, 20% entre 26 et 35SA et plus de 40% avant 24SA [2]. Dans notre étude l'anamnèse infectieuse était positive avec un bilan infectieux parlant dans 59% des cas, et il s'agissait d'une chorioamniotite maternelle dans 8,3% des cas, ce qui rejoint les données rapportées par les auteurs avec un taux de chorioamniotite clinique lors d'une RPM variant de 10% à 36%. Dans leur étude, Ramsey et al. rapportent un taux de chorioamniotite clinique de 36% pour les naissances avant 26SA et 6% pour les naissances à 35-36SA [3]. L'incidence de l'infection néonatale après une rupture des membranes de plus de 24 heures est approximativement de 1%. Lorsqu'ils existent des signes cliniques de chorioamniotite, le risque augmente de 3 à 5% [4]. Un des signes d'appel d'infection néonatale est l'ouverture prolongée de la poche des eaux qui lorsqu'elle est supérieure à 12heures favorise la contamination du liquide amniotique par voie ascendante [5]. Seince et al [6] dans une étude multicentrique, n'ont pas trouvé de différence significative en termes d'IMF selon que le délai de la rupture soit de 6 à 12 h, de 12 à 24 h ou de plus de 24 h. De même, Mozurkeuwich et Wolf [7], dans une méta-analyse étudiant les conséquences infectieuses néonatales selon le délai d'expectative adoptée en cas de RPM à terme, n'ont pas montré de différence significative en terme d'IMF quelque soit le délai adopté. Dans notre série, le risque infectieux global associé à une RPM isolée était évalué à 18% ainsi un nouveau-né parmi 10, né après une RPM isolée, est à risque d'IMF, ce même chiffre a été rapporté par Seo et al en 1992 [8]. La mise des patientes sous antibiothérapie devant toute rupture prématurée des membranes n'est pas automatique et se discute selon les écoles, dans notre étude c'était le cas dans 28% des cas. Dans notre série, la majorité des nouveau-nés étaient asymptomatiques à l'admission, soit dans 76% des cas, et pour les patients symptomatiques la détresse respiratoire était le motif d'admission en réanimation dans 12,8 % des cas et en soins intensifs dans 30% des cas. L'ictère néonatal précoce était noté chez 31,45% des malades avec une souffrance néonatale sur l'Apgar ou des convulsions néonatales dans 11,5% des cas.

## Conclusion

Ce travail souligne le risque important d'IMF associé à une RPM même à terme. Ce risque est d'autant plus important que la rupture est supérieure à 24 heures de temps. Dans la majorité des cas le liquide amniotique est clair et les nouveau-nés sont asymptomatiques à l'admission, ce qui laisse la mise sous antibiothérapie automatiquement de ces nouveau-nés un sujet encore très discutable.
